# The Protective Effects of Trypsin Inhibitor on Hepatic Ischemia-Reperfusion Injury and Liver Graft Survival

**DOI:** 10.1155/2016/1429835

**Published:** 2015-12-13

**Authors:** Lianyue Guan, Hongyu Liu, Peiyao Fu, Zhuonan Li, Peidong Li, Lijuan Xie, Mingang Xin, Zhanpeng Wang, Wei Li

**Affiliations:** ^1^Department of Hepatobiliary-Pancreatic Surgery, China-Japan Union Hospital of Jilin University, Changchun 130033, China; ^2^Department of Liver Surgery, Key Laboratory of Carcinogenesis and Cancer Invasion of Ministry of Education, Zhongshan Hospital of Fudan University, Shanghai 200032, China; ^3^Department of Tumor Therapy, Tumor Hospital of Jilin Province, Changchun 130033, China; ^4^Department of Vascular Surgery, China-Japan Union Hospital of Jilin University, Changchun 130033, China; ^5^Department of Anesthesiology, China-Japan Union Hospital of Jilin University, Changchun 130033, China

## Abstract

The aim of this study was to explore the protective effects of ulinastatin (urinary trypsin inhibitor, UTI) on liver ischemia-reperfusion injury (IRI) and graft survival. We employed mouse liver cold IRI and orthotopic liver transplantation (OLTx) models. UTI was added to lactated Ringer's (LR) solution for liver perfusion and preservation *in vitro* or combined with UTI injection intraperitoneally to the liver graft recipient. Our results indicated that UTI supplementation protected the liver from cold IRI in a dose-dependent manner and prolonged liver graft survival from extended cold preserved liver donors significantly. The underlying mechanism of UTI on liver IRI may be mediated by inhibition of proinflammatory cytokine release, increasing the expression of the antiapoptotic gene *Bcl-2* and decreasing the expression of the proapoptosis genes of *Caspase-3* and *Bax*, and further protects hepatocytes from apoptotic death and improves liver function.

## 1. Introduction

Liver transplantation has become one of the most effective methods for the treatment of end-stage liver disease. However, hepatic ischemia-reperfusion injury (HIRI) remains a challenging issue for liver surgeons; moreover, the pathogenic mechanism of HIRI has not been elucidated completely. HIRI may be initiated by liver cell oxidative stress and may further induce primary graft nonfunction or function failure after liver transplantation. In addition, HIRI limits the applications of marginal donor livers. An effective therapeutic method for preventing HIRI is still lacking. Ulinastatin (urinary trypsin inhibitor, UTI), a trypsin inhibitor, plays an important role in inhibiting proinflammatory cytokine release. The application of UTI has been long-standing in clinical practice. It has been shown that UTI has certain protective effects against liver injury. However, the mechanisms are still not very clear and remain to be further investigated. The objectives of this study were to explore the protective effects of UTI against HIRI, the effects of UTI on liver cell oxidative stress, and the impact of UTI on graft survival, as well as the underlying mechanisms.

## 2. Materials and Methods

### 2.1. Animals

Male C57BL/6 (B6; H2b) mice, 8–14 weeks of age, were purchased from Beijing Weitong Lihua Laboratory Animal Technology Co., Ltd. (Beijing, China) and maintained in a modified pathogen-free facility of Jilin Surgical Research Institute at China-Japan Union Hospital of Jilin University. The mice were provided with Purina Rodent Chow and tap water* ad libitum*. Animal care was in compliance with our institutional animal care and use committee-approved protocol and with the “Guide for the Care and the Use of Laboratory Animals” published by the National Institutes of Health.

### 2.2. Reagents

UTI was purchased from Guangdong Techpool Biochemical Pharmaceutical Co., Ltd. (Guangzhou, China). Lactated Ringer's Solution was obtained from Baxter Healthcare Corporation (Deerfield, IL, USA). Rat anti-mouse IL-6, TNF-*α*, and IFN-*γ* antibodies were from BD Pharmingen (San Diego, CA, USA), rat anti-mouse IL-10 antibody was from Abcam (Cambridge, UK), Bcl-2, caspase-3, and Bax, *β*-actin were from Cell Signaling Technology, Inc., (Danvers, MA, USA), and the ApopTag Peroxidase In Situ Apoptosis Detection Kit was from the Millipore Corporation (Billerica, MA, USA).

### 2.3. UTI Treatment Protocol

The livers were flushed via the portal vein with 5 mL of cold LR or UW solution containing heparin 20 units/mL with or without UTI supplementation of 10–10000 U/mL and then were procured and stored in a sterile container containing 2 mL (equal mouse total body blood volume) perfusion solution for 6 hours. Alanine aminotransferase (ALT), aspartate aminotransferase (AST), and lactate dehydrogenase (LDH) enzyme release from the preservative solution and the liver histology were examined, respectively. In the orthotopic liver transplantation study, the donor livers were preserved in the LR solution at an extended time of 1 hour, with or without UTI supplementation at 1000 U/mL, respectively. Liver biopsy and functional assays were performed at 1 hour, 6 hours, and 18 hours posttransplantation, respectively.

### 2.4. Orthotopic Liver Transplantation

Orthotopic liver transplantation (OLTx) with revascularization was accomplished with a combination of suture and cuff techniques performed between syngeneic strain combinations as described [1, 2]. The donor livers were preserved in the LR solution at an extended time of 1 hour, with or without UTI supplementation at 1000 U/mL, respectively. The liver graft recipients were also received subcutaneous injection of UTI 1000 U in 0.2 mL LR or an equal volume of LR solution immediate after complication of liver transplantation, respectively. Graft function, liver cell apoptosis, and expression of inflammatory cell factors were evaluated posttransplantation.

### 2.5. Liver Enzyme Assays

Samples from the liver preservation solutions at different concentrations of UTI supplementation at 6 hours in the LR solution and in the serum at 1, 6, and 18 hours posttransplantation were assayed for ALT, AST, and LDH levels using the Beckman Coulter Synchron UniCel DXC800 automatic analysis system, respectively.

### 2.6. Histological Analysis

Formalin-stored tissue specimens were embedded in paraffin and cut into 4 *μ*m sections and were then examined by routine hematoxylin and eosin (H&E) staining. Three samples from each group and 10 high power-fields of each sample were analyzed. The histology scores of the liver tissue sections were determined by 2 independent persons in a blind manner according to the following scoring criteria: 0, no hepatocellular damage; 1, mild injury characterized by cytoplasmic vacuolization and focal nuclear pyknosis; 2, moderate injury with dilated sinusoids, cytosolic vacuolization, and blurring of intercellular borders; 3, moderate to severe injury with coagulative necrosis, abundant sinusoidal dilation, RBC extravasation into hepatic chords, and hypereosinophilia and margination of neutrophils; 4, severe necrosis with loss of hepatic architecture, disintegration of hepatic cords, hemorrhage, and neutrophil infiltration. Criteria to specifically evaluate polymorphonuclear leukocyte (PMN) infiltration (0, zero; 1, minimal; 2, mild; 3, moderate; 4, severe) were also used [3].

### 2.7. TUNEL Staining

Apoptotic cells in paraffin sections (10 *μ*m) were identified using the ApopTag Peroxidase In Situ Apoptosis Detection Kit (Millipore) according to the manufacturer's instructions, as described [1]. The sections were first deparaffinized, then fixed for 10 min at room temperature (RT) in 10% neutral buffered formalin (pH 7.4), followed by two washes (5 min each) in phosphate buffered saline (PBS). Endogenous peroxidase activity was quenched in 2% H_2_O_2_, before exposure to terminal deoxynucleotidyl transferase (TdT) enzyme at 37°C for 60 min. After washing in stop wash buffer (37°C, 30 min), anti-digoxigenin-peroxidase was added (RT; 30 min). Diaminobenzidine (DAB) (ScyTek Laboratories, Inc., Logan, UT, USA) was used for color development, and the sections were counterstained with hematoxylin. The numbers of apoptotic cells in the liver sections were counted under the light microscope by the numbers of apoptotic cells per 40 high-power fields in five sections per tissue per mouse (three mice per group).

### 2.8. Western Blot

The apoptosis-associated protein Bcl-2, caspase-3, and Bax expression in the liver tissues were determined by the Western blot assay. Proteins (50 *μ*g/sample) extracted from the liver tissue in SDS loading buffer (50 mM Tris, pH 7.6, 10% glycerol, 1% SDS) were subjected to 12% SDS-polyacrylamide gel electrophoresis and transferred to a PVDF membrane (Bio-Rad Laboratories, Inc., Hercules, CA, USA). The gel was then stained with Coomassie blue to document protein loading. The membrane was blocked with 5% dry milk and 0.1% Tween 20 (Bio-Rad) in PBS. The membrane was subsequently incubated with the primary antibodies at 4°C overnight. The primary antibodies were mouse monoclonal anti-human Bcl-2, caspase-3, and Bax antibody (Cell Signaling Technology). The membranes were developed according to the Amersham Enhanced Chemiluminescence protocol. Beta-actin was measured as a loading control.

### 2.9. Immunohistochemistry Staining

The paraffin sections of the liver tissues were stained for tumor necrosis factor alpha (TNF-*α*), interleukin (IL)-6, IL-10, and interferon-gamma (INF-*γ*) expression by the avidin-biotin-peroxidase complex (ABC) method, as described [1]. The paraffin sections were deparaffinized, followed by trypsin incubation to restore the antigen. Endogenous peroxidase activity was quenched in 2% H_2_O_2_. Purified rat anti-mouse TNF-*α*, IL-6, IL-10, and IFN-*γ* antibodies (Abs) were applied (BD Pharmingen) overnight, followed by biotin-anti-rat immunoglobulin G (IgG). Rat IgG was used as isotype control. ABC (Vector Laboratories, Inc., Burlingame, CA, USA) was then added. DAB (ScyTek) was used as the substrate, and sections were counterstained with hematoxylin. The results were counted automatically under a high-power microscope (400x). The cytokine levels were analyzed using ECL Plus image lab software (Life Technology, GeneSnap, USA) and numerically expressed by the ratio of positive cells versus whole cells per high-power field. The data were averaged from 10 high-power fields for each section and 3 samples from each group.

### 2.10. Statistical Analysis

Statistical analysis was performed using SPSS version 22.0 software (IBM Corporation, Armonk, NY, USA). The results are expressed as mean ± SE. Comparison between groups was made by one-way analysis of variance (ANOVA), and data counts were analyzed by the chi-square test. Survival data were analyzed by the Kaplan-Mayer log-rank test. Probability (*P*) values < 0.05 were considered statistically significant.

## 3. Results

### 3.1. UTI Supplementation of the LR Perfusion and Preservation Solution Reduced the Liver Enzyme Release of ALT, AST, and LDH Significantly in a Dose-Dependent Manner

**Figure 1:**
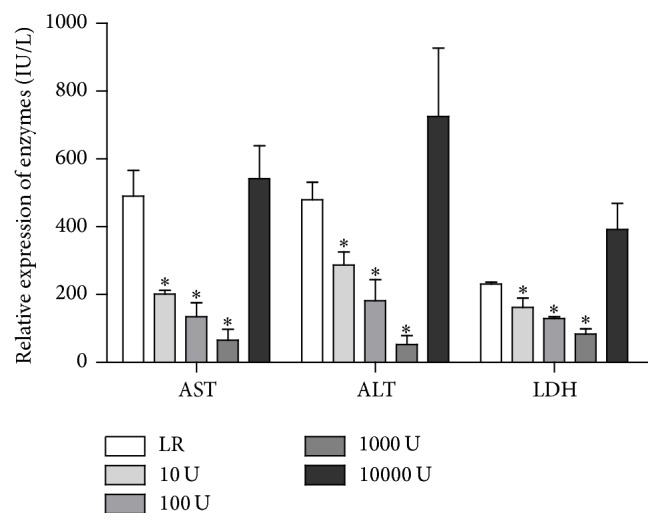
UTI supplementation to the perfusion and preservative LR solutions significantly inhibited liver enzyme release. UTI was added to LR solution at concentration of 10–10000 U/mL; the livers were perfused with 5 mL LR solution with or without UTI supplementation and were then procured and stored in a sterile container containing 2 mL (equal mouse total body blood volume) perfusion solution for 6 hours. The samples were pooled from 3 livers in each group; then, the ALT, AST, and LDH enzyme levels were measured. UTI concentration of 1000 U/mL showed maximal effects. Compared to LR control, ^*∗*^ *P* < 0.05. The results are representative of 3 separate experiments.

We first examined whether UTI supplementation of LR solution can protect the liver from cold ischemia injury. B6 mouse livers were perfused with 5 mL of cold Ringer's solution containing heparin 20 units/mL with or without the addition of UTI (10–10000 U/mL). The livers were then preserved in 2 mL of the above solution on ice for 6 hours. ALT, AST, and LDH levels from the preservation solution were tested from 3 livers pooled together in each group. The levels of ALT, AST, and LDH were detected to have significantly increased after 6 hours of cold preservation in the LR control group. The release of ALT, AST, and LDH was reduced significantly in the UTI-supplemented group compared to the LR-only group. The concentration with optimal efficacy was 1000 U/mL, while an excessive concentration (10000 U/mL) aggravated HIRI (Figure 1). Similarly, we tested UTI supplementation of UW solution at a concentration of 1000 U/mL. The release of ALT, AST, and LDH was reduced significantly in the UTI-supplemented group after 18 hours of preservation (data not shown), indicating that UTI has a uniformly protective effect on the liver.

### 3.2. UTI Supplementation of LR Solution Alleviates the Degree of Liver Tissue Damage and Prolongs Liver Graft Survival after an Extended Ischemia Time

**Figure 2:**
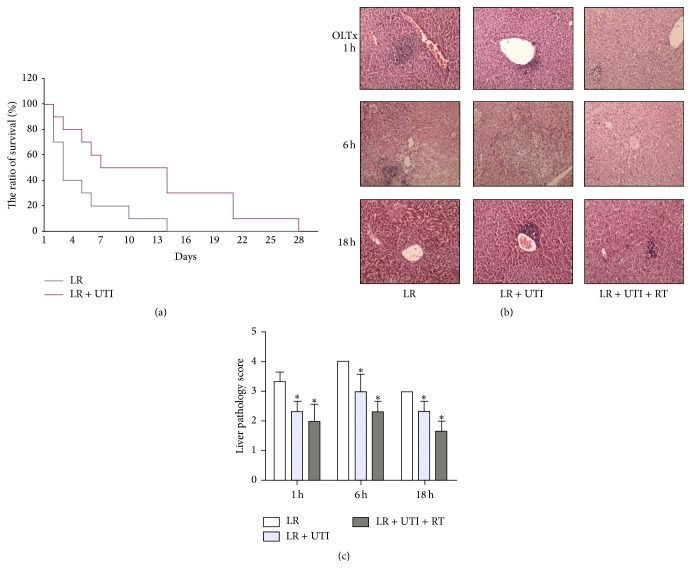
(a) Liver graft survival from extended cold-preserved donors. Syngeneic orthotopic liver transplantation was performed between inbred C57BL/6 strain mice. The liver donors were preserved* in vitro* in cold LR solution with or without UTI addition at 1000 U/mL for 1 hour. Liver graft survival was followed posttransplantation. Mean survival time was 9.8 ± 2.44 days in the UTI-treated group (*n* = 10) versus 4.3 ± 1.45 days in control (*n* = 10), ^*∗*^ *P* < 0.05. (b) Liver histology examination was performed by H&E staining at 1, 6, and 18 hours posttransplantation, respectively (200x). UTI alleviated the degree of liver injury and preserved the cell morphology of the extended-time cold-preserved grafts significantly. Increased hepatocyte swelling, increased cytoplasmic vacuolization, nuclear pyknosis, sinusoidal dilatation, and focal necrosis in the LR-only group with maximal injury at 6 h were observed. (c) Liver pathology scores were evaluated from the H&E sections of (b). The data are from 3 samples in each group and 3 separate experiments.

To examine the protective role of UTI on hepatocytes during an extended time course of cold ischemia and the impact on liver graft survival from an extended time course of cold preservation, syngeneic orthotopic liver transplantation (OLTx) was performed in which the liver donors experienced an extended cold preservation time course in LR solution with or without the addition of UTI for 1 hour. The survival rate of the liver grafts in both groups was significantly affected by an extended time course of cold preservation. However, in comparison with the LR group, the survival rate of the liver grafts was significantly improved in the group with UTI supplementation; the 3- and 7-day survival rates were 80% and 50% in the UTI-supplemented group versus 40% and 20% in the LR control group (*P* < 0.05) (Figure 2(a)). Histological examination of H&E sections of liver tissues harvested at 1, 6, and 18 hours posttransplantation revealed that hepatocyte swelling, increased cytoplasmic vacuolization, nuclear pyknosis, sinusoidal dilatation, and focal necrosis developed as early as 1 hour posttransplantation, became worst at 6 hours, and showed restoration at 18 hours posttransplantation. The addition of UTI to the LR preservation solution or combination treatment to the liver graft recipients gave the livers significant protection from cold ischemia injury during the extended preservation period. The liver morphology remained much better in those groups (Figure 2(b)). The overall pathological scores in the LR + UTI and LR + UTI + RT groups were much lower than the scores in the LR control group (Figure 2(c)).

### 3.3. UTI Significantly Decreased the Serum ALT, AST, and LDH Levels of Liver Graft Recipients with Extended Cold Preservation Time of Donor Livers

**Figure 3:**
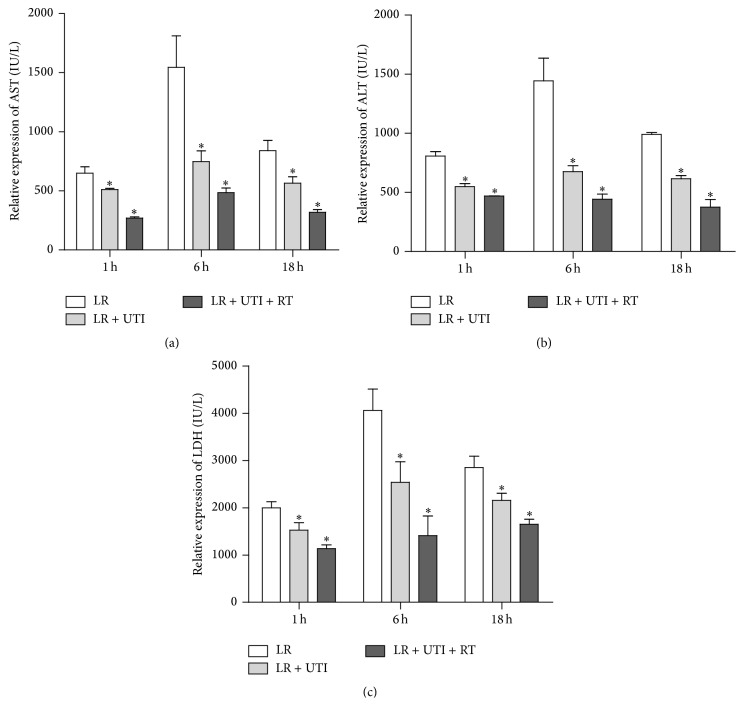
Change in serum enzymes of AST, ALT, and LDH after 1 h, 6 h, and 18 h from liver transplantation. The serum enzyme indicators of each group increased significantly and peaked at 6 h after transplantation. Release of AST, ALT, and LDH was remarkably reduced in the UTI preservation group and the UTI preservation combined with recipient UTI administration group, compared with the control group (^*∗*^ *P* < 0.05).

Liver grafts experience both cold and warm IRI during the process of transplantation. In order to prove the protective effects of UTI on liver grafts, we applied LR solution supplemented with UTI (1000 U/mL) as the cold preservation solution for liver perfusion. The donor livers received extended time course cold preservation for 1 h in LR solution at 4°C, as well as intraoperative subcutaneous injection of UTI 1000 U (16 U/kg in 0.2 mL). The ALT, AST, and LDH levels in the recipient serum were detected, respectively, at 1 h, 6 h, and 18 h after transplantation. The results indicated that the serum enzyme indicators of each group increased significantly at all three time points and peaked at 6 h after transplantation. Release of AST, ALT, and LDH was remarkably reduced in the UTI preservation solution (LR + UTI) group and the combined UTI preservation plus recipient UTI administration (LR + UTI + RT) group compared to the LR control group (*P* < 0.05). In addition, the LR + UTI + RT group showed a minimum degree of enzyme increase, indicating that UTI is capable of protecting the structural integrity of hepatic cells and mitochondria and inhibiting the release of various enzymes of hepatic cells when HIRI occurs (Figure 3).

### 3.4. UTI Supplementation Inhibited Hepatocyte Apoptosis and Modulated Caspase-3, Bcl-2, and Bax Protein Expression

**Figure 4:**
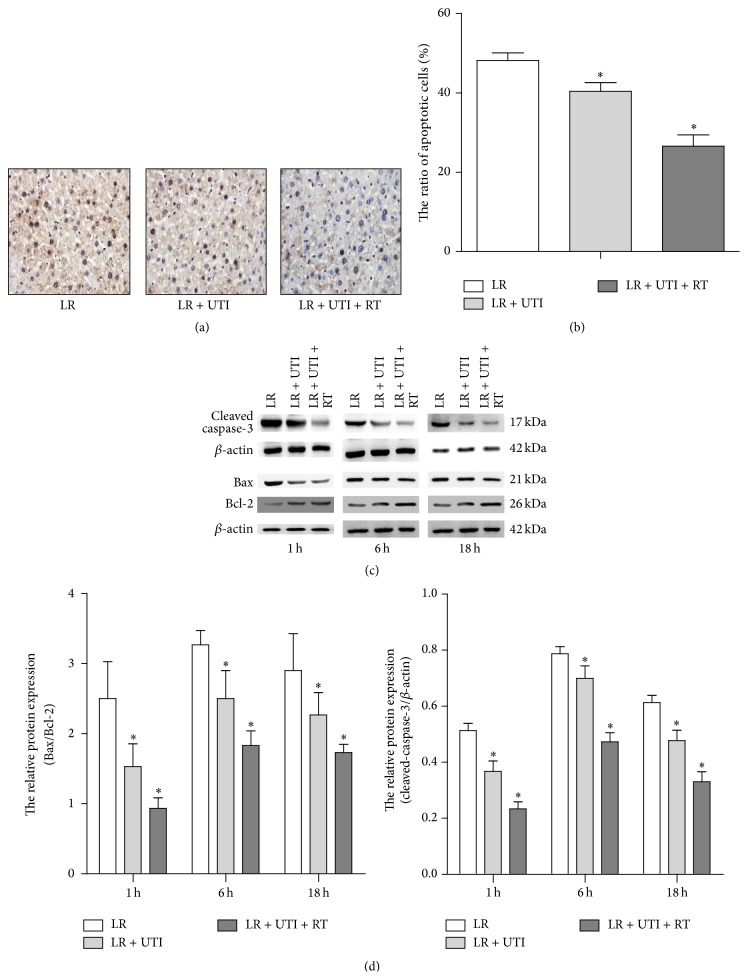
Hepatocyte apoptosis,* Caspase-3*,* Bcl-2,* and* Bax* gene expression on liver tissue. (a) Hepatocyte apoptosis TUNEL evaluation revealed a significantly reduced number of hepatocellular apoptotic nuclei treated with UTI at 6 hours, especially in the combined treatment group (48.2 ± 1.9 versus 40.4 ± 2.2 versus 26.6 ± 2.9), ^*∗*^ *P* < 0.05. (b) In terms of percentage calculation, three specimens in each group were stained under a 400x light microscope and 4–10 positive fields were observed in each section. The mean ratio of positive cells (brown-yellow stained cells) accounting for total cell count in each group was taken. (c)* Caspase-3* and* Bax* gene expression on liver tissue was reduced, but* Bcl-2* was increased significantly in the UTI-treated group, tested by Western blot. (d) The ratios of Bax/Bcl-2 were 1.5 ± 0.3 versus 0.9 ± 0.2 versus 2.5 ± 0.5, 2.5 ± 0.4 versus 1.8 ± 0.2 versus 3.3 ± 0.2, and 2.3 ± 0.3 versus 1.7 ± 0.1 versus 2.9 ± 0.5. Cleaved-caspase-3/beta-actin was 0.4 ± 0.04 versus 0.2 ± 0.02 versus 0.5 ± 0.02, 0.7 ± 0.04 versus 0.5 ± 0.03 versus 0.8 ± 0.03, and 0.5 ± 0.04 versus 0.3 ± 0.04 versus 0.6 ± 0.03 in the LR + UTI, LR + UTI + RT, and LR groups at 1 h, 6 h, and 18 hours after OLTx, respectively. (^*∗*^ *P* < 0.05).

To determine the effects of UTI on liver cell apoptotic activities under the condition of cold ischemia, the liver tissue sections from the recipients with extended cold preserved liver grafts were examined at 6 h after transplantation by TUNEL staining. Our results revealed that the apoptosis rate of the hepatocytes in the UTI supplemented groups was markedly reduced compared with the LR control (*P* < 0.05) (Figures 4(a) and 4(b)). To further analyze the mechanism of UTI on reducing liver cell apoptosis, caspase-3, a cysteine protease critical for executing apoptosis, Bax, a factor to promote apoptosis, and Bcl-2, a family of proteins demonstrated to reduce apoptosis, were measured in liver tissue at 1, 6, and 18 hours after transplantation, respectively. The expression of the proapoptotic gene* Caspase-3* in the LR control group was significantly higher than that in the LR + UTI and LR + UTI + RT groups at all 3 time points after transplantation. In contrast, the anti-apoptotic gene* Bcl-2* showed relatively high expression in the two experimental groups. These results indicate that UTI effectively inhibits the apoptosis of liver cells through promoting the expression of* Bcl-2* and inhibiting the expression of the* Caspase-3* gene (Figures 4(c) and 4(d)).

### 3.5. UTI Significantly Inhibited the Production of Inflammatory Cytokines in the Liver Grafts

**Figure 5:**
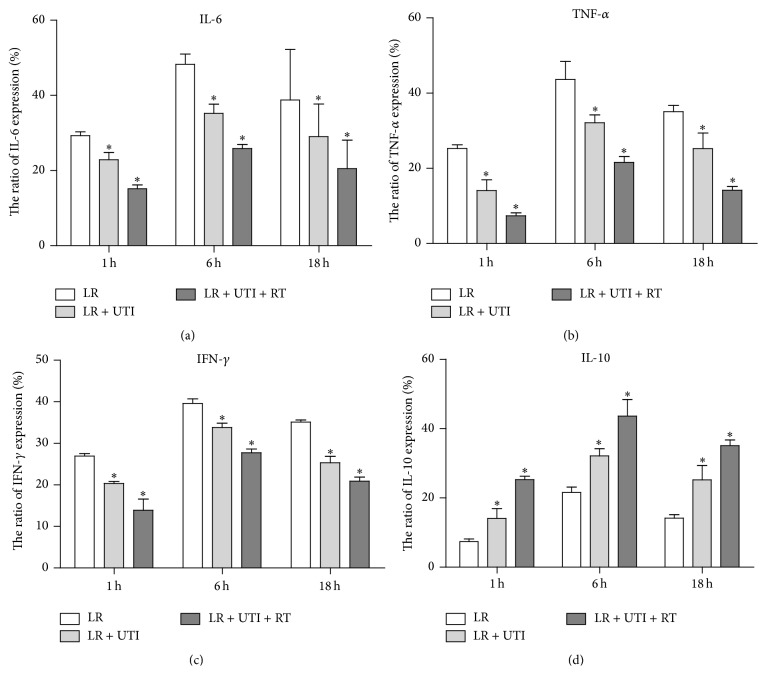
Cytokine levels in the liver grafts at 1 h, 6 h, and 18 h after OLTx. Production of proinflammatory cytokines IL-6, TNF-*α*, and IFN-*γ* in the liver tissues of the UTI-supplemented LR solution with extended cold preservation time was reduced, and IL-10 was increased significantly at 1, 6, and 18 hours after OLTx. ^*∗*^ *P* < 0.05. The data were pooled from 3 samples in each group.

To better understand the mechanisms of UTI on HIRI, expression of proinflammatory and anti-inflammatory cytokines in extended cold-preserved liver grafts was examined by immunohistochemical staining at 1, 6, and 18 hours posttransplantation, respectively. Expression of the proinflammatory cytokines IL-6, TNF-*α*, and IFN-*γ* decreased significantly in the LR + UTI and LR + UTI + RT groups at all three time points posttransplantation compared to that in the LR control group (Figures 5(a)–5(c)). In contrast, the expression of the anti-inflammatory cytokine IL-10 was significantly higher in the LR + UTI and LR + UTI + RT groups than in the LR control group (Figure 5(d)). Overall, UTI inhibited the production and release of proinflammatory cytokines and promoted the expression of anti-inflammatory cytokines, which contributed at least in part to the protection of the liver against cold IRI.

## 4. Discussion

HIRI is a complicated pathophysiological process affected by multiple factors. UTI, as a broad-spectrum protease inhibitor, inhibits multiple proteases including trypsin, *α*-chymotrypsin, hyaluronidase, plasmin, and more [4]. UTI is also capable of inhibiting the release of inflammatory mediators, stabilizing lysosomal membrane, removing harmful free radicals, and inhibiting myocardial inhibitory factor [5]. UTI has been used clinically to treat acute or chronic pancreatitis, severe infection, and acute organ failure. It has also been proved to be an effective perioperative treatment in liver surgery [6–8]. However, the role of UTI in liver transplantation has been seldom reported. Therefore, it is worthwhile to investigate the effects of UTI on liver cold IRI and graft survival from extended cold-preserved donor livers.

The results of the present study showed that the levels of the liver enzymes AST, ALT, and LDH were significantly increased at 6 hours of cold preservation and that supplementation with UTI in either LR or UW preservation solution effectively reduced liver enzyme release. The best protective concentration of UTI was 1000 U/mL, indicating that UTI protects the liver from IRI in a dose-dependent manner. Similarly, in the liver transplantation experiments using the donor livers that experienced extended cold preservation time* in vitro*, the addition of UTI significantly reduced the serum enzyme levels of the liver graft recipients posttransplantation. The maximum reduction in enzyme levels was shown in the group of liver graft recipients who received the combined UTI treatment during the perioperative period. Moreover, liver graft survival was significantly prolonged in the UTI-treated groups. Our results demonstrated that UTI plays a protective role against liver IRI, both in LR and in UW solution, suggesting that UTI may be a useful reagent to extend the cold preservation time of donor livers and offers therapeutic potential to recover more marginal donor livers for organ transplantation.

The protective effects of UTI on liver IRI were directly evidenced by histopathological changes. In comparison with the control group, UTI not only protected the liver cell morphology from cold ischemic injury but also reduced hepatocyte apoptosis and promoted the repair of liver tissue at 18 hours after reperfusion. This suggests that the protective effects of UTI may occur through stabilizing the lysosomal and cell membranes, maintaining the integrity of the vascular endothelial cells, reducing inflammatory cell infiltration, and preventing liver cell apoptosis.

The occurrence of apoptosis is regulated by several pathways, including the caspase-3 and Bcl-2 family members. Caspase-3 is the most important terminal shear enzyme in the process of cell apoptosis and is an important component of the killing mechanism of the cytotoxic T cells. Many studies have shown that the expression of caspase-3 increases significantly when the liver undergoes IRI, which could activate endonucleases and cause liver cell apoptosis by degrading nucleoproteins and activating the release of cytochrome C [9–11]. The Bcl-2 family includes many members, such as the* Mcl-1*,* Bcl-w*,* Bcl-x*,* Bax,* and* Bak* genes, which have either anti- or proapoptosis functions.* Bax* is one of the* Bcl-2* family members that are involved in cell apoptosis, while* Bcl-2* can reduce the release of cytochrome C induced by* Bax* and control apoptosis. Currently, the* Bax/Bcl-2* ratio is usually used to portray the relationship between cell survival and apoptosis; the lower the ratio is, the more significant the anti-apoptosis effects are [12]. The results from our experiments indicate that the* Bax/Bcl-2* and caspase-3/*β*-actin ratios were significantly decreased at the different time courses of 1, 6, and 18 hours after OLTx in the LR + UTI and LR + UTI + RT groups than in the LR control group, indicating that the mechanisms of UTI in protecting the liver from IRI may occur through upregulating the expression of* Bcl-2* and downregulating the expression of* Caspase-3*. The mechanism by which UTI protects the liver from IRI is also attributed to the functions of the antioxidative and stabilizing mitochondrial membrane of UTI [5].

The liver is an organ that produces many cytokines that are involved in a complex regulation network during HIRI. Our study has demonstrated that the cytokines IL-6, IL-10, IFN-*γ*, and TNF-*α* are all involved in the HIRI process. UTI treatment significantly inhibits the production of IL-6, IFN-*γ*, and TNF-*α* but increases the production of IL-10 in the liver at all time courses after liver transplantation. We presume that UTI regulates the production of inflammatory factors in liver tissue after HIRI, resulting in overexpression of anti-inflammatory cytokines, which provides protective effects against damage to the liver tissue. Other studies have also indicated that the protective effects of UTI against liver IRI are probably mediated by inhibiting TNF-*α* and IL-6 through inhibition of the oxidative stress response at the early stage of reperfusion and the activation of monocytes [13]. As we know, the serum cytokine level may more accurately reflect the degree of inflammation in transplant recipients. In order to appropriately elucidate the role of UTI in the regulation of inflammatory cytokines and chemokines, further investigation is needed on the recipient serum cytokine network.

The present study proves that UTI as a supplement added to cold preservation solution for donor livers and perioperative treatment to liver graft recipients both exert strong effects in protecting the liver from the deleterious effects of cold IRI and prolong liver graft survival in recipients who receive extended cold-preserved donor livers. Although our study had the limitations of small sample size and short follow-up time, the protective effects of UTI on HIRI are evidenced by inhibition of liver enzyme release, reduction of the liver pathology score, promotion of anti-inflammatory cytokine production, and decrease in liver cell apoptosis. Studies on other types of protease inhibitors, such as the ubiquitin-proteasome system (UPS) and secretory leukocyte protease inhibitor (SLPI), have shown that these protease inhibitors also have promising therapeutic potential of inhibiting cardiac and liver IRI through the inhibition of IL-1 and TNF-*α*, increase of NO generation, and control of neutrophil extracellular trap (NET) generation [14, 15].

Despite the limitations of the present study, the results are very encouraging. At the least, the study outcome demonstrates the great potential of future application of UTI in liver surgery and the use of marginal donor livers in living donor liver transplantation.

## Abbreviations

Bcl: B-cell lymphoma
DC: Dendritic cell
IFN-*γ*: Interferon-gamma
IL: Interleukin
IRI: Ischemia-reperfusion injury
LR: Lactated Ringer's solution
OLTx: Orthotopic liver transplantation
TNF-*α*: Tumor necrosis factor-alpha
UTI: Ulinastatin.
